# Movement Disorders in Brazil: the seminal contributions of Luiz Augusto Franco de Andrade and Egberto Reis Barbosa

**DOI:** 10.1590/0004-282X-ANP-2020-0388

**Published:** 2021-05-01

**Authors:** Hélio Afonso Ghizoni Teive, Henrique Ballalai Ferraz, João Carlos Papaterra Limongi, Gustavo Leite Franklin, Francisco Eduardo Costa Cardoso

**Affiliations:** 1 Universidade Federal do Paraná Hospital de Clínicas Departamento de Medicina Interna Curitiba PR Brazil Universidade Federal do Paraná, Hospital de Clínicas, Departamento de Medicina Interna, Serviço de Neurologia, Unidade de Distúrbios do Movimento, Curitiba PR, Brazil.; 2 Universidade Federal de São Paulo Escola Paulista de Medicina Departamento de Neurologia São Paulo SP Brazil Universidade Federal de São Paulo, Escola Paulista de Medicina, Departamento de Neurologia, Unidade de Distúrbios do Movimento, São Paulo SP, Brazil.; 3 Universidade de São Paulo Hospital das Clínicas Departamento de Neurologia São Paulo SP Brazil Universidade de São Paulo, Hospital das Clínicas, Departamento de Neurologia, Unidade de Distúrbios do Movimento, São Paulo SP, Brazil.; 4 Universidade Federal de Minas Gerais Hospital das Clínicas Departamento de Medicina Interna Belo Horizonte MG Brazil Universidade Federal de Minas Gerais, Hospital das Clínicas, Departamento de Medicina Interna, Serviço de Neurologia, Unidade de Distúrbios do Movimento, Belo Horizonte MG, Brazil.

**Keywords:** History of Medicine, Neurology, Movement Disorders, Basal Ganglia Diseases, História da Medicina, Neurologia, Transtornos dos Movimentos, Doenças dos Gânglios da Base

## Abstract

The major advances in the area of movement disorders in Brazil in recent years were driven by the work of Luiz Augusto Franco de Andrade and Egberto Reis Barbosa. This historical review describes the contributions made by these researchers, physicians, and educators to the development of this field in Brazil.

## RESUMO

Os maiores avanços observados na área de distúrbios do movimento nos últimos anos no Brasil teve como fator catalizador a atuação exponencial dos professores Luiz Augusto Franco de Andrade e Egberto Reis Barbosa. Esta revisão histórica enfatizou as contribuições desses pesquisadores, médicos e professores para o desenvolvimento da área no Brasil.

Movement Disorders, among the different sub-specialties of Neurology, has grown exponentially worldwide in recent years^1^^,^^2^. The interest in movement disorders (previously defined as disorders of the extrapyramidal system) emerged in Brazil through the research of Antonio Austregésilo in Rio de Janeiro^3^^,^^4^, and increased by the continuous scientific support of Andrew Lees, mainly regarding events of continuing education^5^^,^^6^. Luiz Augusto Franco de Andrade (at that time, at the Escola Paulista de Medicina [EPM], currently the Universidade Federal de São Paulo) and Egberto Reis Barbosa (Hospital das Clínicas, Universidade de São Paulo) played a crucial role in the development and advance of studies on this topic in Brazil.

## LUIZ AUGUSTO FRANCO DE ANDRADE

**Figure 1:**
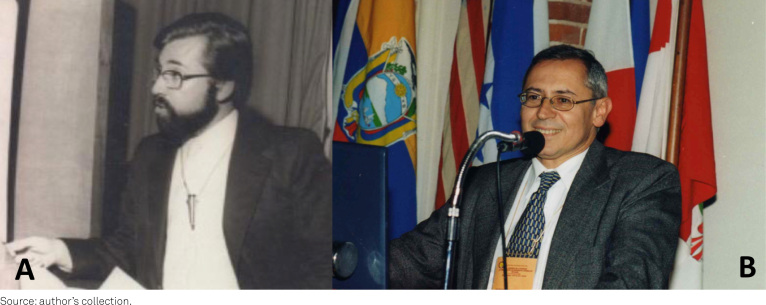
Professor Luiz Augusto Franco de Andrade (A) and Professor Egberto Reis Barbosa (B) early in their careers.

**Figure 2:**
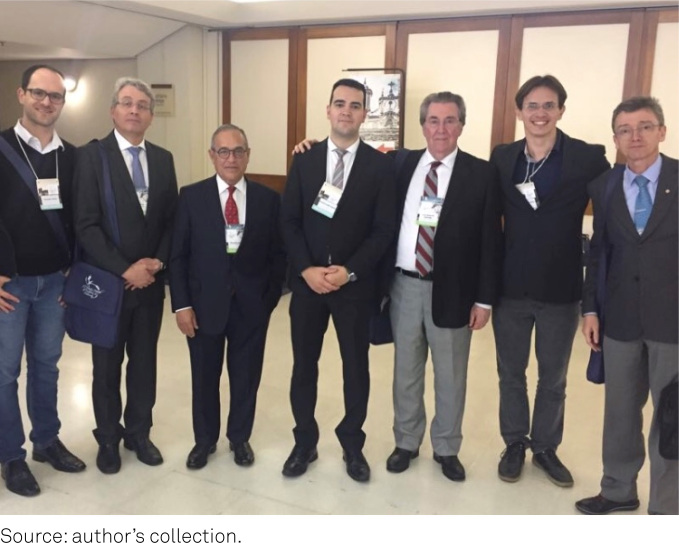
From left to right: Dr. F. Tensini, Dr. H. Teive, Dr. O. Gershanik, Dr. G. Franklin, Prof. Luiz Augusto Franco de Andrade, Dr. A. Meira and Dr. F. Cardoso) in the 11th National Meeting of Movement Disorders, held in Ouro Preto, Brazil, 2018.

Luiz Augusto Franco de Andrade was born in the city of Marília, São Paulo, in 1944, but settled in Londrina, in the state of Paraná, where he finished his elementary and high school studies^7^. He entered the Federal University of Paraná School of Medicine in 1963, graduating in 1968, and subsequently completed his medical residency in Neurology at EPM in 1971. In 1973 he completed a fellowship in the area of cerebrovascular diseases, under the supervision of John Marshal at the Queen Square Institute of Neurology, in London^7^. He returned to Brazil in 1973 and was hired at the Neurology Service at EPM^7^. His involvement with Parkinson's disease and movement disorders started in 1978, when he received a request from the head of the Department of Neurology to review the topic “Neurochemistry and Neurotransmitters” and to present a lecture for the members of the Neurology Service at EPM. Based on this presentation, Dr. Elisaldo L. Carlini, Professor of Psychopharmacology at EPM, invited Andrade to develop his PhD thesis in the Department of Psychobiology, under his supervision. At that time, Professor Carlini was investigating the pharmacological effects of cannabis on behavior and sleep^8^. His hypothesis was that REM sleep deprivation could induce dopamine supersensitivity^9^. In 1982, Professor Andrade presented his PhD thesis entitled “REM sleep deprivation in an experimental model of Parkinson's disease in rats’ under the supervision of Professor Carlini^7^^,^^10^. From 1978 onwards, Andrade coordinated the Extrapyramidal Disease Unit at EPM and, since that time, this service became a national reference in the area of movement disorders^7^. In 1985, in a partnership with Roberto Melaragno, Andrade coordinated the first international symposium on movement disorders, which was held in the Brazilian cities of São Paulo and Rio de Janeiro, and involved renowned experts such as David Marsden (UK), Oscar Gershanik (Argentina) and Carlos Chouza (Uruguay)^7^ (Figure 1A). In 1995, he completed his post-doctoral studies with a thesis entitled “Contribution to the study of early-onset parkinsonism: clinical and therapeutic considerations”^7^. He was an Associate Professor of Neurology at EPM from 1978 to 1997, and head of the Neurology Service at EPM from 1995 to 1997. Andrade has continued to participate in many events throughout Brazil, Latin America, and abroad^7^, where his vast expertise in movement disorders was always evident, as well as his broad knowledge and experience in the field of general Neurology. Andrade participated in the creation of the Neurology Recycling and Investigation Study Group (GERIN), which organizes continuing education events about movement disorders across Brazil. Also, he has edited three books, numerous chapters, and has published 74 scientific manuscripts^7^^,^^10^^,^^11^. Finally, it is necessary to highlight his enthusiastic and generous support of younger colleagues, certainly one of the main factors taken into account for the thriving of movement disorders in Brazil (Figure 2).

## EGBERTO REIS BARBOSA

**Figure 3:**
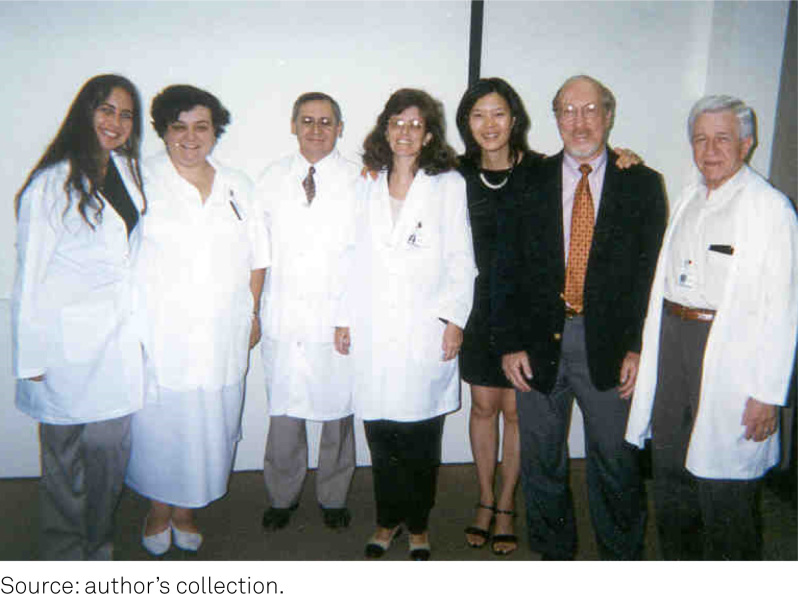
From left to right: Dr. L. Gonçalves, Dr. M. Haddad, Prof. Egberto Reis Barbosa, Dr. M. Gonçalves, Dr. S. Chien, Prof. H. Singer, Prof. Aron Diament.

Egberto Reis Barbosa was born in the city of Mirassol, São Paulo in 1948^12^. He carried out his elementary and high school studies in the towns of Brotas and São José do Rio Preto, respectively, in the state of São Paulo. He graduated in 1973 and did his Medical Residency in Neurology at FMUSP's Hospital das Clínicas from 1974 to early 1976, when he was invited to coordinate the outpatient clinic for Extrapyramidal Diseases at the same hospital^12^. At that time, he was mentored by Professor Horacio M. Canelas, who was the head of the Neurology Service at FMUSP and was engaged in the study of Wilson's disease. Professor Canelas suggested that Barbosa should join the recently established Outpatient Clinic for “Abnormal Movement Diseases”. Afterwards, he completed his Master's Degree in 1986 and his PhD in 1990, at FMUSP^12^, and was appointed a collaborator Instructor of Medicine at the same institution in 2000. Since he began coordinating the Abnormal Movements Clinic in 1976, Professor Barbosa has participated in trainings in the field of countless doctors not only from Brazil, but also from many Latin America countries^12^. He also held several leadership roles at FMUSP's Hospital das Clínicas and other hospitals in São Paulo. In addition, he developed a high-level line of research on different types of movement disorders, particularly on Wilson's disease, in which he is considered a worldwide reference^12^^,^^13^^,^^14^. The cohort of patients with Wilson's disease who are followed up at FMUSP's Outpatient Movement Disorders Clinic is one of the largest in the world^14^. He is, perhaps, among the few clinicians of the world who have personally examined and followed up over one hundred patients with Wilson's disease. Barbosa has been part of the GERIN since 1998 and has participated in continuous education events in the movement disorders around the world (Figure 1B). He also has published several books, book chapters, and more than 200 scientific papers.^11^^,^^12^^,^^13^^,^^14^ In several of the previously mentioned educational endeavors, he has partnered with Andrade, which led them to achieve the status of father figures for the subsequent generation of physicians in the field of movement disorders (Figure 3).

With their work in several hospitals and medical schools, Luiz Augusto Franco de Andrade and Egberto Reis Barbosa have been driving forces in the area of movement disorders, and they are responsible for the development and major advances seen in this field in recent years in Brazil.
